# Synthesis of mono Cytochrome P450 in a modified CHO-CPR cell-free protein production platform

**DOI:** 10.1038/s41598-024-51781-6

**Published:** 2024-01-13

**Authors:** Jan Felix Knauer, Christian Schulz, Anne Zemella, Doreen A. Wüstenhagen, Ruben Magnus Walter, Jan-Heiner Küpper, Stefan Kubick

**Affiliations:** 1 Fraunhofer Project Group PZ-Syn of the Fraunhofer Institute for Cell Therapy and Immunology, Branch Bioanalytics and Bioprocesses (IZI-BB), Potsdam, Germany; 2 Fraunhofer Institute for Cell Therapy and Immunology (IZI), Branch Bioanalytics and Bioprocesses (IZI-BB), Potsdam, Germany; 3 Freie Universität Berlin, Institute of Chemistry and Biochemistry – Biochemistry, Berlin, Germany; 4 Institute of Biotechnology, Brandenburg University of Technology Cottbus-Senftenberg, Senftenberg, Germany; 5 Institute of Biotechnology, Technische Universität Berlin, Gustav-Meyer-Allee 25, 13355 Berlin, Germany; 6 Faculty of Health Sciences, Joint Faculty of the Brandenburg University of Technology Cottbus –Senftenberg, the Brandenburg Medical School Theodor Fontane and the University of Potsdam, Potsdam, Germany

**Keywords:** Biochemistry, Biotechnology, Drug discovery, Molecular biology

## Abstract

Cytochromes P450 (CYPs) are a group of monooxygenases that can be found in almost all kinds of organisms. For CYPs to receive electrons from co-substrate NADPH, the activity of NADPH-Cytochrome-P450-oxidoreductase (CPR) is required as well. In humans, CYPs are an integral part of liver-based phase-1 biotransformation, which is essential for the metabolization of multiple xenobiotics and drugs. Consequently, CYPs are important players during drug development and therefore these enzymes are implemented in diverse screening applications. For these applications it is usually advantageous to use mono CYP microsomes containing only the CYP of interest. The generation of mono-CYP containing mammalian cells and vesicles is difficult since endogenous CYPs are present in many cell types that contain the necessary co-factors. By obtaining translationally active lysates from a modified CHO-CPR cell line, it is now possible to generate mono CYPs in a cell-free protein synthesis process in a straightforward manner. As a proof of principle, the synthesis of active human CYPs from three different CYP450 gene families (CYP1A2, CYP2B6 and CYP3A4), which are of outstanding interest in industry and academia was demonstrated. Luciferase based activity assays confirm the activity of the produced CYPs and enable the individual adaptation of the synthesis process for efficient cell-free enzyme production. Furthermore, they allow for substrate and inhibitor screenings not only for wild-type CYPs but also for mutants and further CYP isoforms and variants. As an example, the turnover of selected CYP substrates by cell-free synthesized CYPs was demonstrated via an indirect luciferase assay-based screening setup.

## Introduction

In humans, the superfamily of Cytochrome P450 (CYP) enzymes comprises 18 families of heme-containing proteins that belong to the group of oxidoreductases. Enzymes of only three CYP families are involved in hepatic drug metabolism. The name CYP450 derives from its characteristic light absorbance attribute that is caused by the inherent heme group^1^. Due to their almost unique properties, reaction mechanism and long evolutionary history, these enzymes have been studied intensely in various aspects since their discovery^2,3^. These studies had great practical benefits and allow for example the use of most drugs in medicine^4^.

Eukaryotic CYPs can be found in different cell organelles, mainly in the endoplasmic reticulum^5,6^. Since CYPs are the only human enzymes capable of catalyzing hydroxylation of non-activated carbon atoms, they have a very broad and overlapping substrate specificity and additionally they form a variety of isoforms^7^. In hepatocytes, CYPs mainly process xenobiotics, thereby conducting the first step of their body excretion^8^. Many drugs, which are ultimately also xenobiotics, are also metabolized by CYPs. The importance of CYPs for drug development and validation is therefore far-reaching and has already been described in detail in various reviews^9–11^. Due to individual CYP polymorphism, CYPs are also an important factor in the implementation of individualized medicine, particularly in the dosage of pharmaceuticals^12^. The majority of hepatically metabolized drugs involves the CYP enzymes CYP1A2, CYP2B6, CYP2C9, CYP2C19, CYP2D6 and CYP3A4/5 accounting for more than 79% of drug oxidation^13,14^. Some drugs are designed as prodrugs to be bioactivated by CYPs in order to form an active component^15^. On the other hand, CYPs significantly determine the half-life of certain individual drugs. In both cases, the drug concentration in an organism is highly dependent on CYPs. Additionally, drugs themselves can act as activators and inhibitors for CYPs. Therefore, interference of the drugs with the enzymatic pathway of CYPs must be considered. Consequently this interference may result in malfunction of the metabolism mechanisms and therefore leading to severe side-effects^14^.

Since CYPs have an extensive influence on drug metabolism, a reliant screening mechanism with specific CYPs for drug discovery would facilitate the investigation of the metabolic fate of novel drugs as well as of CYP-inhibitors and modulators. CYP inhibition as well as induction can lead to failures of several drugs and a consequent withdrawal from the market. This issue has also been addressed in a comprehensive publication^14^. To circumvent such issues, screenings can be performed by using human liver cell microsomes from primary hepatocytes, representing the physiological CYP spectrum or more CYP specific by using microsomes derived from genetically modified cells that only express one CYP, so called mono CYP microsomes. The usage of primary hepatocyte microsomes for CYP analysis as well as drug metabolization studies bears the disadvantage to address all variants of endogenous CYPs simultaneously, thereby impeding mono CYP analysis. The recombinant expression of CYPs has already been extensively investigated^16^. However, mammalian CYPs in particular are difficult to produce. Restrictions are caused by the complex co-factor requirements, like the heme group and the availability of the redox partner, cytochrome P450 oxidoreductase (CPR)^17^.

Bacterial systems such as *Escherichia coli* are probably the most popular platform for recombinant protein expression, due to their straightforward handling and high growth rate. However, the synthesis of complex eukaryotic proteins like CYPs is unfavorable since they can only be expressed in a modified soluble form^16,18^. In contrast to prokaryotes, yeast as well as higher eukaryotes like insect and mammal cells, possess organelles like the endoplasmic reticulum and the Golgi apparatus, enabling proper anchoring of membrane bound proteins. Usually the redox partner NADPH-Cytochrome-P450-oxidoreductase (CPR) is co-expressed to ensure CYP activity^19–21^. Additionally the co-expression of the chaperon led to an increased yield of active protein for some CYPs by supporting the folding mechanism^22^. The co-expression of auxiliary factors also plays a role likewise in prokaryotic and in eukaryotic recombinant expression systems.

For industrial purposes a common expression host for recombinant eukaryotic CYPs is *Saccharomyces cerevisiae*, for example for the large scale production of the antimalarial artemisinin through the coexpression of a CPR, CYP71AV1 from *Artemisia annual* and other enzymes^23^. Also, mammalian CYPs have been used to design a biosynthetic pathway, including 4 CYPs, in yeast for the generation of hydrocortisone from simple carbon sources^24^. When it comes to mammalian expression systems several liver cell-lines have been shown to be capable of CYP overexpression. However, these cell lines come with the disadvantage of background CYP activity. This problem can be circumvented by functional overexpression of CYPs together with CPR in CHO or HEK cells as shown in recent studies^25,26^. However, harnessing the advantages of eukaryotic systems for the cell-free synthesis of CYPs remains an unexplored field. Cell-free protein synthesis (CFPS) has the potential for flexible and adjustable analysis of individual CYPs. Taking advantage of endogenous membrane structures eukaryotic cell-free systems have been successfully used for the synthesis of other membrane localized proteins^27^. In contrast to cell-based expression systems, the CYP translation process is directly accessible. This open system can be directly manipulated and allows the straightforward supplementation of additional components to the reaction like heme and heme precursors (δ-aminolevulinic acid, glucose, glycine, as well as different iron species) as well as heme-producing enzymes to receive active CYPs^28^. Additionally, it is possible to modify the cells that are used for lysate production similar to cell-based systems. Hereby, CPR can be integrated into the endogenous microsomes in advance to create the suitable reaction environment for CYPs. The stable modification of eukaryotic CHO cells with CPR has been realized earlier^20^. The desired CYP enzyme can be synthesized in the translationally active modified CHO-CPR lysate in a straightforward manner. Subsequently, various screening assays can be performed without any purification or further processing steps in 96 and 384- well plates. In this context advantages and limits of cell-free synthesis for drug development have been addressed recently^29^.

## Aim of the work

Cytochrome P450 enzymes are one of the best-studied classes of enzymes, but recombinant production is challenging due to their membrane localization and enzymatic coupling. The use of vesicle-based cell-free protein synthesis, which enables the fast and efficient production of various membrane proteins, can provide an alternative way of producing defined active human CYPs. The aim of this study is to outline a protein synthesis platform that enables the synthesis of all kinds of CYPs within only a few hours. For this purpose, modified CHO cell lysates containing the necessary CYP co-factors were generated and characterized. In these lysates, CYPs from different gene families were synthesized. CYP1A2, CYP2B6 and CYP3A4 are prominent representatives of the three most important human CYP families and are therefore utilized as a proof of concept in this study. Finally, cell-free synthesized CYPs are used to demonstrate the straightforward applicability of the system for screening procedures.

## Methods

### Template generation

Templates for the synthesis of CYPs in cell-free systems were generated by Biocat GmbH. The protein encoding sequence and further regulatory factors for CAP-independent protein synthesis by using a Cricket paralysis virus-IRES^30^ (Gene number 714916-1/2/3, 724709-12) was integrated in a pUC57-1.8k-vector backbone.

### Cell fermentation, lysis and lysate procession

Suspension adapted Chinese Hamster Ovary cells (CHO-K1) were routinely cultivated in ProCHO5 medium (Lonza Group AG, Basel, Switzerland) supplemented with 6 mM l-alanyl-l-glutamine (Merck, Darmstadt, Germany). CHO suspension cells were cultured in non-baffled flasks (Corning, New York, USA) at 37 °C and 5 vol-% CO_2_ at 100 rpm on an orbital shaker. CHO cells were grown in suspension cultures in shaking flask to a maximal volume of 500 mL or in a 5 L bioreactor. CHO cells were harvested at a density of approximately 4 × 10^6^ cells/mL. During incubation in the fermenter, viability, oxygen concentration, pH and cell density were monitored. Cell washing, lysis and lysate processing were performed as described earlier^27,31,32^. In short, cells were centrifuges at 200×*g* for 10 min, and the pellet washed with 40 mM HEPES–KOH (pH 7.5), 100 mM NaOAc and 4 mM DTT. The pellet was then resuspended in the same buffer at a density of approximately 5 × 10^8^ cells/mL. Cell-disruption was performed by syringing the suspension through a 20-gauge needle. After a final centrifugation step at 10,000×*g* for 10 min the supernatant was applied to a size-exclusion chromatography column (Sephadex G-25, GE Healthcare, Freiburg, Germany) and elution fractions with high RNA content were pooled. Residing mRNA was digested by addition of 10 U/mL micrococcal nuclease S7 (Roche, Mannheim, Germany) and 1 mM CaCl_2_. After incubation for 2 min 6.7 mM EDTA (f.c.) were added. Finally, the lysate was immediately shock-frozen and stored at − 80 °C.

Lysates were prepared from CHO-K1 cells. Additional to the CHO-K1 wild type cell line, lentiviral modified CHO cells that either express human CPR (CHO-CPR) or human CPR together with CYP3A4 (CHO-CPR/CYP3A4) were used. Blasticidin (Biovision GmbH, Ilmenau, Germany) (3 µg/mL, resistance of the CPR expression vector) or Blasticidin and Zeocin (Abcam, Cambridge, UK) (300 µg/mL, resistance of the CYP3A4 expression vector) were added to the culture medium, to maintain the expression of human CPR or CPR/CYP3A4 in the corresponding CHO cell lines. The lysis process for the generation of translationally active lysates was the same as for wild type CHO-K1 cells.

### Cell-free protein synthesis

Synthesis of proteins in translationally active lysates derived from cultured Chinese hamster ovary (CHO) cells and its modified variants CHO-CPR and CHO-CPR/CYP3A4 cells, was performed in batch based systems as previously described^27^. Accordingly designed, plasmids suitable for cell-free protein synthesis (CFPS), coding for the CYP of interest, were applied as template. T7-RNA-Polymerase, amino acids, an energy regeneration system and other supplements were added to the translationally active lysates with the additional supplementation of 5 µM heme (porcine) (Alfa Aesar Haverhill, Massachusetts, USA) to the reaction and a reaction temperature of 24 °C was set unless noted otherwise. For the isolation of microsomes the translation mixture (TM) was centrifuged at 16,000×*g* for 10 min at 4 °C. The pellet was resuspended in the same volume of PBS to receive the microsomal fraction (MF). The microsomal fraction comprises the endogenous microsomes derived from the endoplasmic reticulum including the de novo synthesized membrane bound proteins.

### Protein yield determination

To validate successful cell-free protein synthesis, radioactive labeling of de novo synthesized proteins with ^14^C-leucine was performed enabling qualitative characterization by autoradiography and quantitative analysis through scintillation counting as described earlier^33^. Disintegrations per minute (dpm) were measured by liquid scintillation counting performed using the Hidex 600 SL (Hidex). Protein yields were calculated based on the dpm, the molecular weight of the synthesized protein, the specific radioactivity A_spec_ (Eq. 1) and the total number of leucines in the target protein (Eq. 2).

1 $${A}_{spec}= \frac{{c}_{14C-Leu} \cdot {A}_{14C-Leu Stock}}{{c}_{total Leu}}$$

2 $$concentration\left[\frac{\upmu g}{mL}\right]=\frac{measured counts \left[\frac{dpm}{mL}\right]\cdot molecular weight \left[\frac{\upmu g}{pmol}\right]}{{A}_{spec}\left[\frac{dpm}{pmol}\right]\cdot {\#}_{leucines in the protein}}$$

### Acetone precipitation, SDS-PAGE and Autoradiography

Sodium dodecyl sulfate polyacrylamide gel electrophoresis (SDS-PAGE) and autoradiography were used to analyze homogeneity and molecular weight of in vitro translated proteins. 45 μL water were added to 5 µL of the sample and precipitated with 150 μL ice cold acetone at 4 °C for at least 15 min. Precipitated proteins were pelleted at 16,000 × g for 10 min at 4 °C. Protein pellets were dried for 1 h at 45 °C and re-suspended in 20 μL LDS sample buffer. The samples were loaded onto 10% SDS-PAGE gels. SDS-PAGE was performed at 150 V for 1 h. The gels were stained for 1 h using SimplyBlue—SafeStain, and destained in water over night. The gels were dried (Unigeldryer) for 70 min at 70 °C. The dried gels were put on a phosphor screen for at least three days. Radiolabeled proteins were visualized on the Amersham Typhoon laser scanner (GE Healthcare).

### Western blot

Western blotting and subsequent antibody detection were used for the identification of endogenous and de novo synthesized CYP3A4 and CPR in the translation mixture of the cell-free synthesis reaction. SDS-PAGE was performed like described above. Proteins were blotted on a PVDF membrane with an iBlot device (Thermo Fisher Scientific, Waltham, Massachusetts, USA). The membrane was washed three times with TBS and subsequently blocked with 2% Bovine Serum Albumin (BSA) (Carl Roth GmbH + Co. KG, Karlsruhe, Germany) over night at 4 °C. After three washing steps with TBS/T, the membrane was incubated with the primary antibody at a concentration of 0.4 μg/mL in 2% BSA for three hours at room temperature. The blot was washed three times with TBS/T and incubated with a secondary Horse Radish Peroxidase (HRP) linked antibody at a final concentration of 0.5 μg/mL in 2% BSA at room temperature for one hour. Three final washing steps in TBS/T were performed. Chemiluminescent signals were detected after incubation with ECL detection reagent. The primary antibody used for the detection of CPR was “CYPOR (F-10): sc-25270” (Santa Cruz Biotechnology, Dallas, Texas, USA), the primary antibody used for the detection of CYP3A4 was “CYP3A4 (HL3): sc-53850” (Santa Cruz Biotechnology, Dallas, Texas, USA).

### Fluorescence microscopy

Confocal laser scanning microscopy was used to analyze protein translocation. In preparation, the microsomal fraction was separated from the rest of the translation mixture as described above. 5 µL of the MF were diluted in 15 µL PBS and transferred on chambered Coverslips (ibidi GmbH, Gräfelfing, Germany), The samples were analyzed by confocal laser scanning microscopy using a LSM 510 Meta (Zeiss). Therefore, samples were excited with an argon laser at 488 nm, and the emission signals were recorded with a bandpass filter in the wavelength range from 505 to 550 nm. Photobleaching was performed using an argon laser at 488 nm with 100% laser intensity. After photobleaching pictures were taken each minute for 14 min.

### CPR activity assay

CPR activity was determined by the NADPH dependent conversion of the water-soluble tetrazolium salt WST-8 using the “Cytochrome P450 Reductase Activity Assay Kit” (Abcam, Cambridge, UK). The assay was performed according to the manufacturers protocol. The activity was determined directly in the translationally active lysate of wild type (wt) CHO cells and CHO-CPR cells. Additionally, the microsomal fraction was isolated as described previously. The activity was quantified using a calibration curve that was generated with supplements supplied by the kit.

### CYP activity assays

For CYP activity measurement, “P450-Glo™ Assays” (Promega, Madison, Wisconsin, USA) were used. CYP1A2 activity was detected by Methoxy-Luciferin (Luciferin-ME) turnover (V8772), CYP2B6 was detected by Dimethoxybutyl-Luciferin (Luciferin-2B6) turnover (V8321) and CYP3A4 was detected by Luciferin isopropyl acetal (Luciferin-IPA) turnover (V9001). The reaction was performed according to the Promega “P450-Glo™ Assays” protocol except the CYP reaction time was prolonged to 1 h unless otherwise noted. The reaction temperature was set at 37 °C. The NADPH Regeneration System (V9510) was used for the supply of NADPH during the assay. Three control approaches were performed, one with buffer control, one designed as no template control and one control, using human liver cell microsomes (Gibco™ Human Microsomes, 50 Donors) (Thermo Fisher Scientific, Waltham, Massachusetts, USA) as positive control. Human microsomes were tested at a final concentration of 0.4 mg/mL. If not otherwise noted 5 µL of the microsomal fraction of the cell-free reaction were applied as samples to the activity assay. For response condition adjustments, CYP activities were usually expressed as percentage of the highest CYP activity during the assay. For CYP activity quantification, a standard curve was prepared using beetle luciferin (Promega, Madison, Wisconsin, USA) according to the protocol.

### Indirect substrate screening

Luciferase-based assays were also used as a preliminary screening procedure for the turnover of various potential CYP substrates. The substrates testosterone (Sigma-Aldrich Chemie GmbH, Taufkirchen, Germany), midazolam (Sigma-Aldrich Chemie GmbH, Taufkirchen, Germany), efavirenz (Fisher Scientific GmbH, Schwerte, Germany), and phenacetin (Fisher Scientific GmbH, Schwerte, Germany) were solved at a concentration of 3 mM in 100% methanol. Cholesterol as a steroid that is not known to be a CYP substrate of the selected CYPs, was used as control substrate and was prepared in the same way as the substrates. Cell-free CYP synthesis and isolation of the microsomal fraction was performed as described above. The luciferase based CYP activity assay was performed using 5 µL of the microsomal fraction of the cell-free synthesis of CYP1A2, CYP2B6 and CYP3A4. A final concentration of 200 µM of the analyzed substrate was added to the CYP reaction in parallel to the specific luciferase substrate. A vehicle control with methanol was performed to exclude an influence of the solvent. Changes in the turnover of the luciferase product indicate an interaction of the test substrate with the tested CYP. Changes in luminescence signal were expressed in percentage with reference to the result from the batch without addition of a substrate.

### Statistical analysis

Excel Data Analysis tools were used for statistical analysis, especially to test for statistical significance between two independent samples. After F-test for variance, a variance corresponding t-test was performed for records that were normally distributed.

## Results

CYP3A4 is known to be involved in the metabolism of most approved medications. Consequently, it was selected as a model protein for initiating cell-free synthesis of cytochrome P450s in eukaryotic lysates.

### Generation of a modified CHO-CPR Lysate

Modified CHO-K1 cell-lines were cultivated similar to wild-type CHO-K1 cells described earlier^27^. Using a lentivirus vector system, CHO-CPR and CHO-CPR/CYP3A4 cell clones were generated. A doubling rate of about 48 h of CHO-CPR and CHO-CPR/CYP3A4 compared to the wild type cell line with a doubling rate of about 24 h slowed down the process, but did not prevent the achievement of sufficiently high cell densities. The different cell-lines were harvested in the exponential growth phase. Typical growth conditions in the fermenter are shown exemplarily for CHO-CPR cells (Appendix). To retain translocationally active microsomes in the lysate, cells were mildly disrupted using a 20-gauge syringe. After buffer exchange and supplementation of the raw lysate, translational active lysates of the modified cell lines were prepared similar to the process of wild type cell lysate generation. With total target protein yields of around 40 µg/mL at standard conditions, the protein translation in the modified lysates is in the same range as observed for the typical CHO based cell free protein synthesis. After validation of translational activity, the lysates from CHO-CPR cells and CHO-CPR/CYP3A4 cells were additionally analyzed for their CPR and CYP activity.

### Validation of CPR activity in the generated CHO-CPR-lysates

**Figure 1:**
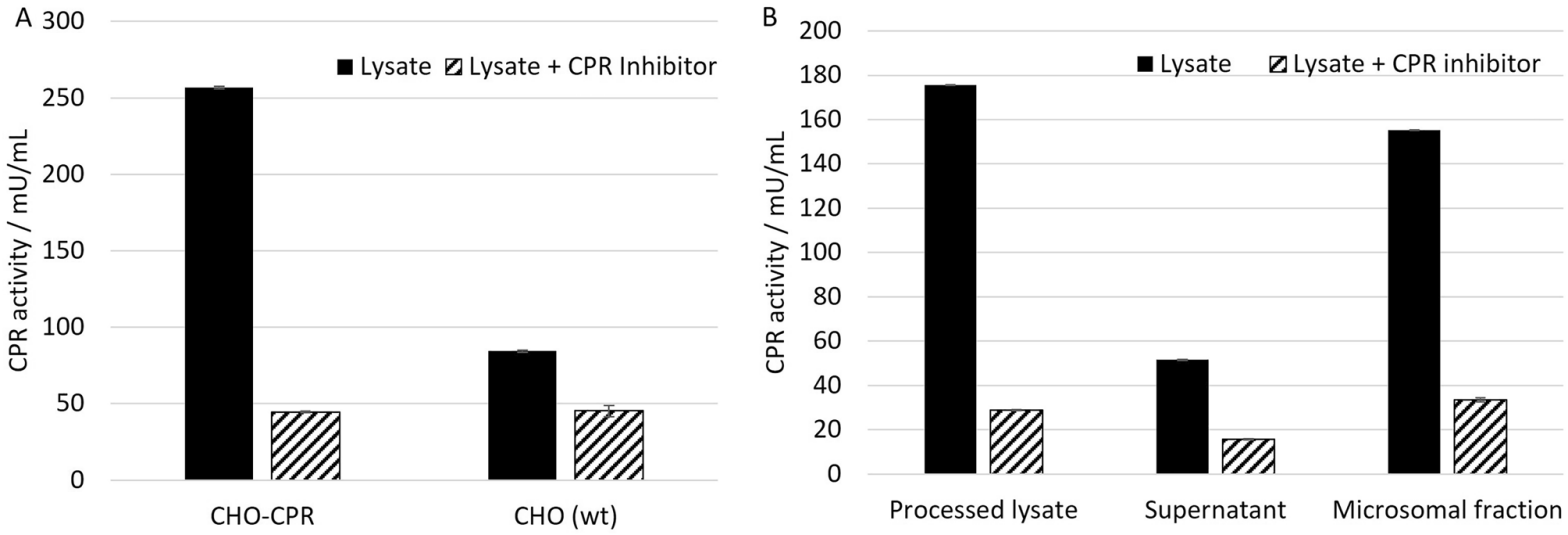
Validation of the CPR activity in the generated lysates by Cytochrome P450 reductase activity assay kit (colorimetric) (ab204704). The assay was performed according to the manufactures protocol. Diphenyleneiodonium chloride was used as inhibitor control. (**A**) CHO-CPR cells were lysed and processed according to protocols for the generation of translationally active lysates. Two lysates were generated: CHO-CPR and CHO-K1 wt cell lysates. (**B**) CHO-CPR lysate was fractionated by centrifugation at 16,000×*g* for 10 min. CPR activity of the supernatant, the microsomal (Pellet) fraction and the processed lysate (lysate before centrifugation) were compared. Standard deviations were calculated from triplicate analysis (n = 3).

CPR acts as co-enzyme for the analyzed CYPs, therefore it is mandatory for their activity. By engineering CHO-K1 cells a more than threefold increased CPR activity could be detected (Fig. 1A). The processed lysates from CHO-CPR cells were centrifuged to separate the endogenous microsomes from the soluble components of the lysate at 16,000×*g* for 10 min. Activity in the CPR-Assay was drastically improved compared to CHO wild type cells. The activity can be detected in particular in the microsomal fraction with about 90% of the total activity in the translation mixture (Fig. 1B). While this increase in activity can be linked to the overexpression of CPR, the residual low activity measured in the supernatant fraction could as well stem from soluble cytoplasmic reductases such as novel reductase^34^. Moreover, the signal in the supernatant might result from non-pelleted smaller vesicles in the CHO system, that need a higher centrifugation speed^35^.

To further characterize the CPR activity of the lysates on CYPs, cell-free synthesized CYP3A4 was produced in the translationally active lysates. CYP3A4 served as model protein for the cell-free synthesis of CYPs.

### Cell-free synthesis of CYP3A4 in CHO-CPR lysates

**Figure 2:**
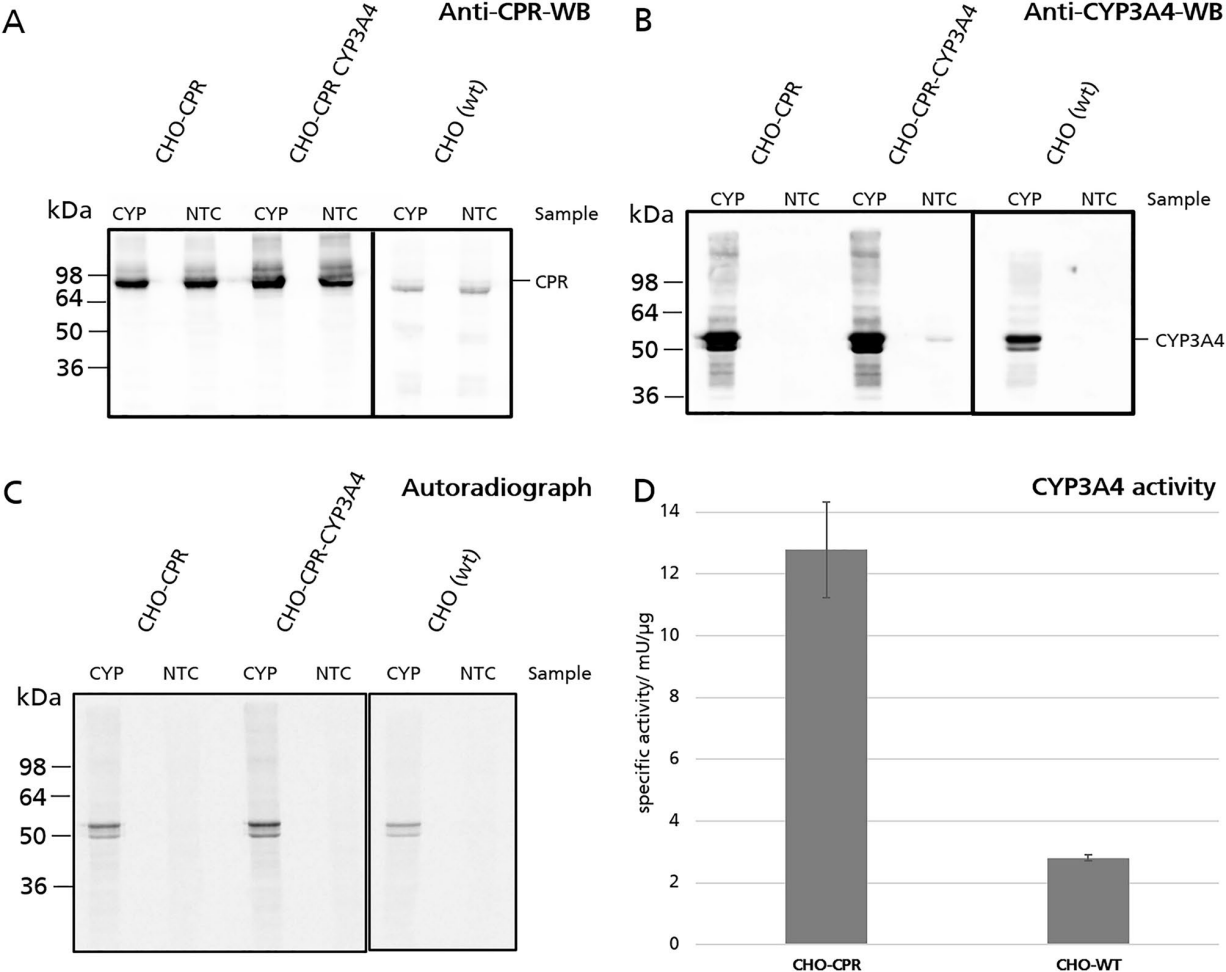
Characterization of translationally active CHO lysates derived from genetically modified CHO cells after cell-free synthesis of CYP3A4. CYP3A4 (57 kDa) and CPR (82 kDa) were identified in the translation mixture through SDS-PAGE (10%) and subsequent western blotting with anti CPR antibodies (**A**) and anti CYP3A4 antibodies (**B**) followed by a secondary HRP linked antibody. Cell-free synthesis of CYP3A4 was compared to no-template-controls (NTC) in each sample. An autoradiograph (**C**) allows the detection of ^14^C-Leucine labeled cell-free synthesized proteins in the cell-free reaction. The activity of CYP3A4 per µg synthesized protein in the different lysates was determined by an IPA-Luciferin assay after CYP3A4 cell-free synthesis. Synthesized protein was quantified through ^14^C-labeling. Background activity from the NTC was subtracted. Measurements were performed as triplicates (n = 3). Blots and autoradiographs visible in each individual sub-image were created simultaneously and treated equally.

CYP3A4 was produced in a batch-based cell-free synthesis. The synthesis was performed using three different lysates: a wild type CHO lysate, a lysate from the aforementioned CPR-expressing CHO cell line and a lysate from CHO cell line expressing CPR as well as CYP3A4. In each lysate a negative control cell-free reaction without the addition of any DNA template (no-template-control = NTC) was performed. The presence of CPR and CYP3A4 in the translation mixture from each batch reaction was visualized via antibody detection on a western-blot (Fig. 2A,B). In the anti-CPR western blot, well-defined bands are detectable at approximately 90 kDa. However, these are less prominent in the wild-type CHO lysate compared to the modified lysates (Fig. 2A). A well-defined band at ~ 55 kDa and a second 50 kDa side-band in the anti CYP3A4 western blot can be detected in any sample where CYP3A4 has been synthesized in a cell-free manner (Fig. 2B). In addition, a much weaker band at ~ 55 kDa can be detected in the NTC of CHO-CPR-CYP3A4 lysates. Besides the western-blot autoradiography was used to visualize to cell-free synthesized CYP3A4 labeled with ^14^C-leucine (Fig. 2C). Similar to the anti-CYP3A4 western-blot, a well-defined band at the level of about 55 kDa with a 50 kDa side-band was detected in the samples containing the DNA template. However, no bands in any NTC were observed.

A CYP3A4 specific luminescent assay (Luminescent Assays and Screening Systems for Measuring CYP Activity (Promega, Madison, USA)) was performed for enzyme activity determination. CHO-WT lysate already shows production of active CYP, while producing a low background signal. This activity could be increased by co-synthesizing CPR in the cell-free reaction (Fig. A2). However, by far the most CYP3A4 activity was measured in CHO-CPR Lysate after cell-free CYP3A4 synthesis, that notably exceeds the activity of CYP3A4 in the CHO-WT-lysate (Fig. 2D). Alternatively, the cell-free synthesis of CYP3A4 in an insect lysate was explored. This led to comparable activities, while exhibiting a higher background signal (Fig. A2).

### Adaptations of reaction conditions

**Figure 3:**
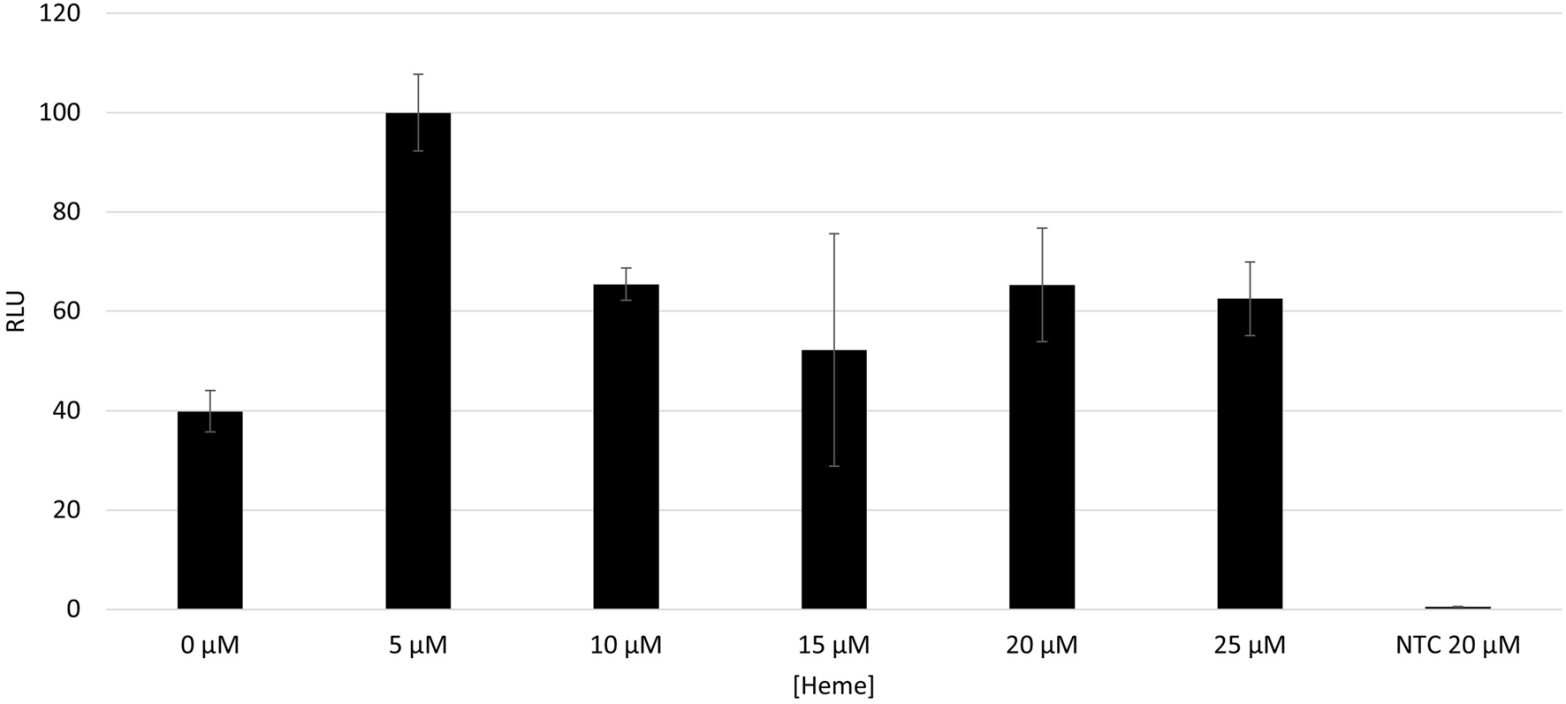
Influence of heme concentration on CYP3A4 activity during cell-free protein synthesis. The synthesis reaction was performed in a batch mode for 3 h. Activity was determined using an IPA luciferase activity assay with a sample size of 2 µL. Standard deviations were calculated from triplicate analysis (n = 3).

Heme is a cofactor of CYPs and is therefore indispensable for its function. Adaption of the amount of supplemented heme to the cell free reaction is therefore mandatory. Heme was supplemented in different concentrations to different batches of the cell-free reaction. The CYP activity in the MF was determined by the Luciferase based CYP3A4 activity assay. A heme concentration of 5 µM resulted in the highest CYP3A4 activity, which was more than twofold higher compared to the control without supplementation (Fig. 3).

The supplementation of higher concentrations of heme results in equally 60% reduced activity compared to the 5 µM heme supplemented sample. The concentration of 5 µM heme was used in all subsequent batches. For an increased CYP activity, the cell-free reaction temperature was adapted to 24 °C instead of 30° usual for CHO-CFPS (Fig. A3).

### Localization, yield and activity of cell-free produced CYP3A4

**Figure 4:**
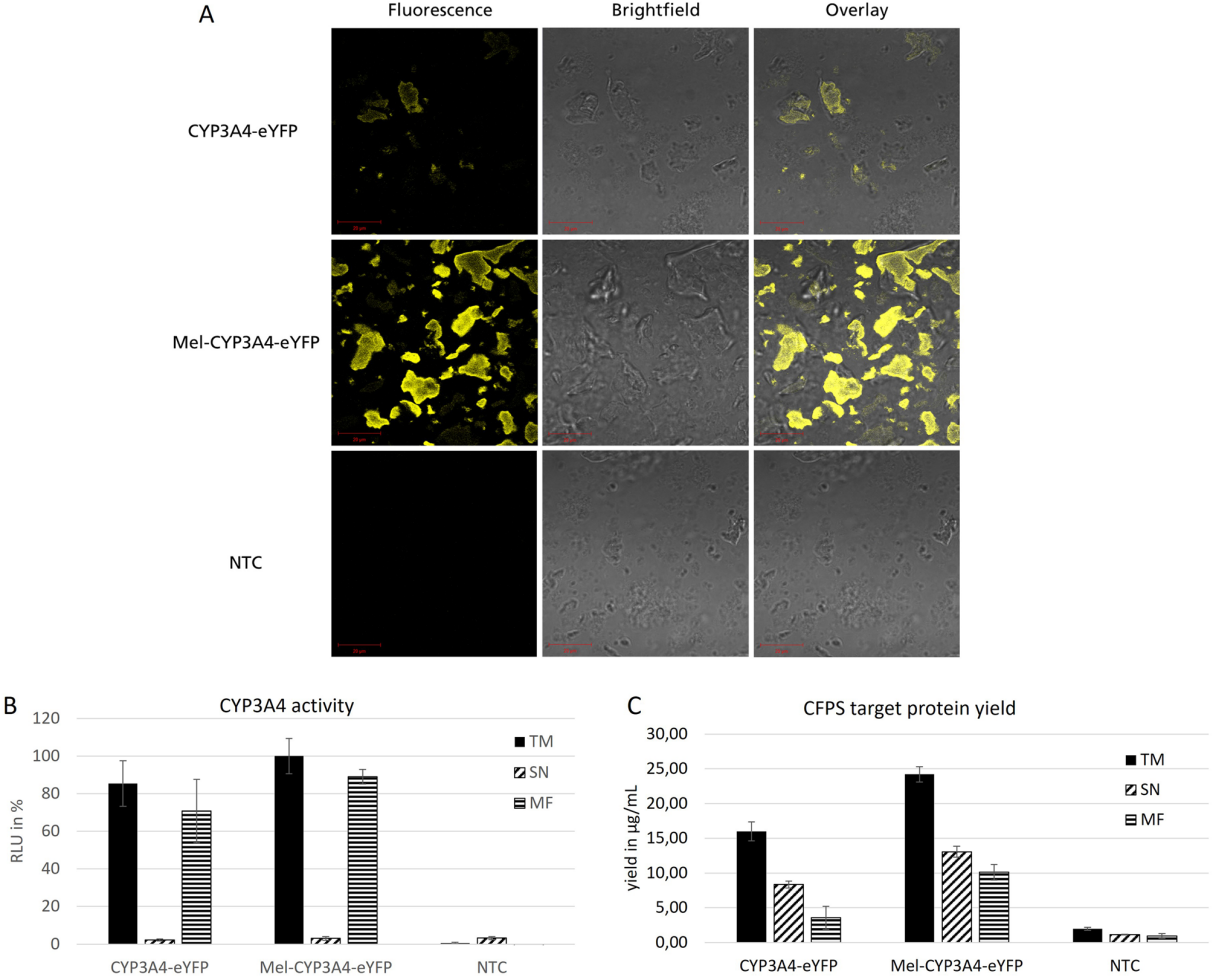
(**A**) Confocal microscopy images of cell-free synthesized CYP3A4-eYFP and Mel-CYP3A4-eYFP. The proteins were synthesized in batch mode for 3 h. The microsomal fraction of the cell-free reaction was analyzed. The fluorescent image, a brightfield image and an overlay of both images are shown. NTC = no template control; translation reaction without DNA template. (**B**) Determination of CYP3A4-eYFP and Mel-CYP3A4-eYFP yield and enzyme activity after cell-free synthesis in the translation mixture (TM), the microsomal fraction (MF) and the supernatant fraction (SN). Enzyme activity was determined by an IPA-luciferase assay (Promega). (**C**) Additionally, the yield of cell-free produced proteins was determined via radioactive labeling followed by TCA precipitation and scintillation counting. Standard deviations were calculated from triplicate analysis (n = 3).

CYPs are membrane associated proteins, but in contrast to most trans-membrane proteins they are only N-terminally anchored to the membrane and have a partially lipophilic surface that is oriented towards the membrane. The translocation process therefore differs from other membrane proteins that have already been produced successfully by CHO-based cell-free protein synthesis (CFPS). Consequently, localization and the influence of signal sequences are an important issue for the cell-free synthesis of CYPs. The localization of the cell-free produced CYPs was analyzed using confocal laser scanning microscopy. For this purpose, templates for CYP3A4-eYFP fusion proteins were generated. Additionally, a template containing a melittin signal sequence upstream of the transmembrane segment (Mel-CYP3A4-eYFP) was generated. Both templates were used for cell-free protein synthesis in the modified CHO-lysates. Fluorescence microscopy reveals a distinct difference of CYPs produced with the Mel containing template compared to the template without the Mel signal sequence. The CYPs harboring the signal sequence are preferentially localized at the endogenous microsomes (Fig. 4A). According to the yield determination, the addition of a melittin signal sequence led to an increased rate of translocation of 40% (Fig. 4C). However, despite the higher protein yields the volumetric activity only increased slightly (Fig. 4B).

**Figure 5:**
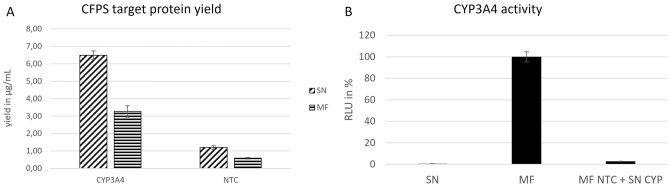
Analysis of posttranslational translocation of CYP3A4. Proteins were synthesized in the batch mode for 3 h. The microsomal fraction was analyzed and compared to the microsomal fraction of an NTC to which the supernatant fraction of the CYP batch was added. (**A**) Yield determination of cell-free produced proteins via radioactive labeling followed by TCA precipitation and scintillation counting. (**B**) Relative activity of CYP3A4 in the supernatant fraction (SN), the microsomal fraction (MF) and in the microsomal fraction of an NTC to which the supernatant fraction of a 3 h CYP batch synthesis was added and incubated for about an hour. 3 µL per well of the samples were applied in the assay Standard deviations for B and C were calculated from triplicate analysis (n = 3).

The co-localization was further analyzed by comparing the microsomal fraction of the CYP-sample with the microsomal fraction of an NTC to which the supernatant fraction of the sample was added and incubated for about an hour. To determine if functional posttranslational translocation into the microsomes in fact was present, this sample was analyzed using fluorescence microscopy (Fig. A4) and the activity assay. Usually, a co-translational translocation would be expected; however, fluorescence microscopy reveals a similar image as in the microsomal fraction. The overall intensity of the fluorescence signal seems to be lower than in the MF. The CYPs of the supernatant fraction are not active despite being co-localized with the microsomes of the NTC batch (Fig. 5B) and despite displaying a higher target protein yield than the CYPs in the microsomal fraction (Fig. 5A).

Since yields of active protein could not be improved the data implies, that a notable amount Mel-CYP3A4 is produced inactive, further experiments were performed using CYP without the melittin signal peptide.

### Synthesis of different CYPs and turnover of pharmaceutically relevant CYP substrates

**Figure 6:**
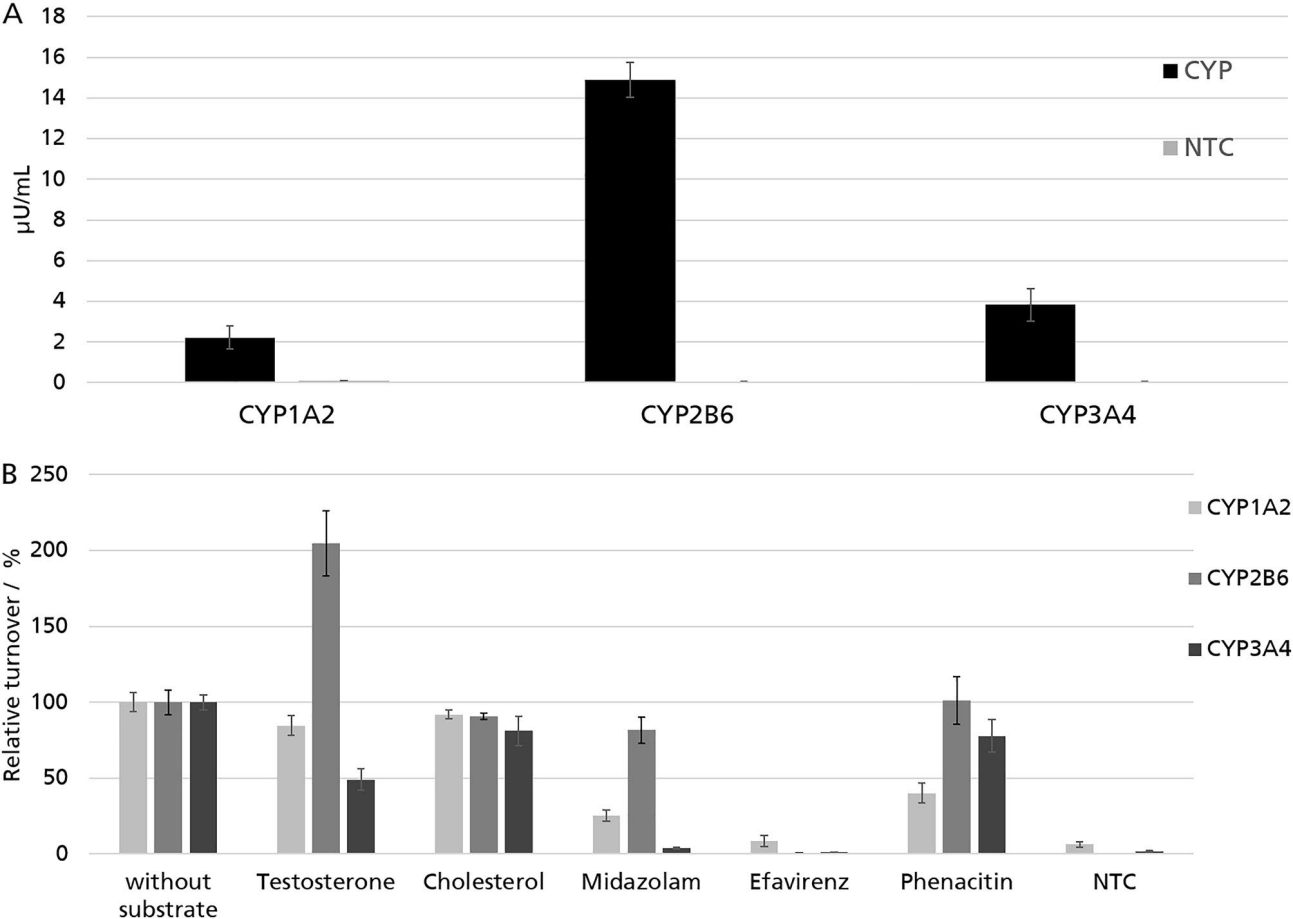
(**A**) Activity determination of different CYPs in the microsomal fraction (MF) after 16,000×*g* centrifugation. (**B**) Screening of different pharmaceutically relevant CYP substrates in relation to a sample without additional substrate. Different CYP substrates were added during the CYP activity assay at a concentration of 200 µM. Cholesterol as a steroid that is not known to be a CYP substrate of the selected CYPs, was used as control substrate. A reduction of Luc signal compared to the vehicle control (without substrate) implies the competitive turnover of the CYP substrate and an inhibition of the CYP activity during the assay. Standard deviations were calculated from triplicate analysis (n = 3).

The application of cell-free protein synthesis enables the time-saving synthesis and analysis of different proteins via template exchange. Besides CYP3A4, CYP1A2 and CYP2B6 were synthesized in the modified CHO cell-free system using the same adapted reaction conditions. Yield determination was executed by scintillation counting of ^14^C-labeled protein. Additional activity assays were performed using the corresponding luciferase based assay (Fig. 6A). All CYPs were active in the microsomal fraction with almost zero background. Cell-free produced CYP2B6 had the highest activity (15 µU/mL) followed by CYP3A4 (4 µU/mL) and CYP1A2 (2 µU/mL).

An indirect activity assay using the luciferase-based assay will identify potential CYP substrates and inhibitors in a screening procedure. For this purpose, various known pharmaceutically relevant CYP substrates (testosterone, midazolam, efavirenz and phenacetin) were used as a proof of principle. CYP1A2, CYP2B6 and CYP3A4 were cell-free synthesized in modified CHO-CPR lysates. Microsomes containing the CYPs were isolated and applied to the corresponding luciferase-based activity assay. CYP substrates to be analyzed were added to the mono-CYP microsomes in the activity assay. All substrates were added at a final concentration of 200 µM each. A sample without additional substrate (vehicle) and an additional sample with cholesterol as non-interacting control substance with the respective CYPs, were prepared as a reference. Due to competitive turnover, adding of CYP substrates should lead to a decrease of luciferase signal due to competitive substrate turnover in batches with interacting substrates (Fig. 6B).

In the competitive assay CYP1A2, CYP2B6 and CYP3A4 were analyzed by their activity changes after adding different CYP substrates. CYP1A2 assay luciferase activity was reduced by all tested substrates while midazolam and efavirenz had the highest impact. CYP2B6 assay luciferase activity reduction was only observed after addition of efavirenz. The supplementation of testosterone led to a 100% increase of monooxygenase activity for CYP2B6. CYP3A4 assay luciferase activity was drastically reduced by testosterone, midazolam and efavirenz.

## Discussion

Recombinant expression of membrane proteins has been challenging for many years^36,37^. More than half of all pharmacologically relevant proteins are membrane-bound^38^. Therefore, an outstanding interest in the development of efficient procedures to produce a wide variety of functional membrane proteins exists. Recent progress in CFPS lead to the successful synthesis of various toxic and membrane bound proteins accessible for research and development^29,39–42^. However, there are only a few studies on CYPs, one of the pharmaceutically most relevant groups of membrane proteins. Recombinant expression of these heme-containing, membrane bound oxidoreductases was attempted frequently in several research studies with some success^16,20^, but partially limited due to the lack of cofactors and a suitable membrane environment, especially for prokaryotic expression systems^28^. However, several commercially available products indicate that there is currently unpublished progress and certainly a demanding interest in CYP production, for example by companies such as Hypha discovery, Xenotech, Merck and Thermo Fisher. Until now, research on cell-free protein synthesis based CYP production is only poorly covered. Cell-free protein synthesis based on vesicle containing eukaryotic cell-extracts allows for the precise development of convenient CYP substrate screening systems. In this context the availability of ER originating and CPR harboring endogenous microsomes, which can be programmed with individual CYPs by cell-free synthesis, is of outstanding advantage.

The electron transfer of CPR is mandatory for the activity of CYPs, therefore, a closer look at CPR localization and activity in translationally active lysates is of fundamental importance^17,43^. Despite CPR activity was detected in the wild type-CHO cells and their lysates per se, an increased CPR activity was detected in CHO lysates derived from the CHO-CPR cell line overexpressing human CPR. The use of CHO cells specifically designed for CYP synthesis and in particular to produce CPR-enriched CHO-CPR lysates, led to a threefold boost of CPR activity due to its overexpression. The microsomes in the CHO lysates originate from the ER of the cells in which CPR and most CYPs are naturally located^44^. Therefore, a natural-like translocation that led to correct localization and folding of CPR in the microsomes can be assumed. Consequently, the generated CHO-CPR lysates are optimally suited for the production of a variety of CYPs. In our study CYP3A4 was used as model CYP for the characterization of the generated CHO-CPR lysates, since it is the most frequently analyzed CYP and responsible for the majority of phase-I xenobiotic and especially drug metabolization in the human liver^11^. Cell-free synthesis of CYP3A4 in the modified lysates led to a fourfold increase of total CYP3A4 activity per volume cell-free reaction compared to synthesis in conventional lysates.

The exploitation of fast and convenient high-throughput screening systems for biomolecules is one of the most remarkable advantages of open cell-free systems^45^. This platform technology enables, for example, the synthesis of different CYPs as well as different CYP variants without time-consuming cloning and fermentation steps^41^. Cell-free protein synthesis based on CHO lysates in this context is a promising technology for various applications, including in vitro drug screening platforms, CYP-specific metabolite phenotyping and synthesis, pharmacologically relevant toxicological studies through to diagnostic applications. The use of CHO cell-lines in protein production is widely established in manifold processes that require a highly evolved eukaryotic expression system. CHO cell-based systems enable the synthesis of complex membrane embedded proteins. For the first time this is shown here in a hybrid model qualifying cell-based and cell-free protein synthesis methods side by side. The lack of CYP background activity in CHO cells^25^ is an additional advantage of this particular cell line for defined CYP applications. Luciferase-based assays are well suited to quantify ratios of CYP activities in different approaches^46^ and quantifications of substrate turnover can additionally be determined by using mass spectrometry^47^. Western blot analysis shows that the amount of cell-free synthesized CYP is significantly higher than in the parallel cell-based approach using CYP-overexpressing CHO-CPR/CYP3A4 cells. To clearly determine if the double band that is observed in the cell-free samples stems from an alternative translation start, a premature termination or has other causes would ultimately need mass spectrometric analysis of the target protein. The difference in the overall synthesis level seems to be even more pronounced than the difference in activity. This may be due to incomplete membrane integration, misfolding or aggregation of a certain amount of cell-free synthesized CYP. Consequently, there is high potential for adaptation of the reaction parameters resulting in the optimal CYP synthesis conditions with further increase CYP specific monooxygenase activity. A prerequisite for an even more efficient synthesis of membrane proteins is a better understanding of the mechanism of translocation in a eukaryotic cell-free protein synthesis system. Translocon interactions and the entire translation process during co-translational translocation, which is essential for the correct localization and the best possible activity of CYPs, are of particular importance^48,49^. Additionally the lipid composition has a significant influence on CYP activity, especially in the context of the enzyme`s hydrophobic substrates^50^.

Besides CPR, heme is the most important co-factor of CYPs, that is mandatory for CYP function^51^. Sufficient availability of heme during cell-free synthesis reaction is of key importance. However, high heme concentration can lead to a decrease in protein activity due to its hydrophobicity and reactivity^52,53^, which also has a negative effect on the total amount of active CYPs. A certain basic concentration of heme might already be present in the cell-free system, since basal CYP activities can be measured even without the further addition of heme^53^. Interestingly above 5 µM a plateau below the optimum is reached. A similar observation was made for the synthesis of unspecific peroxygenases in an insect-cell-free system^54^. Since in both cases the heme supplementation had no influence on the translation efficiency in the analyzed concentration range, there seems to be a more intricate underlying mechanism potentially affecting protein folding. By using confocal microscopy, the co-localization of fluorescently labeled CYPs and microsomes can be detected. An addition of the melittin signal sequence to the template increased the effect of apparent translocation that could be observed during microscopy. The target protein yield in the microsomal fraction determined by radioactive labeling confirms an increased CYP concentration in the microsomal fraction using the melittin signal sequence. This is in accordance to results observed for several other cell-free synthesized secretory and membrane proteins^55,56^. However, the addition of the melittin signal sequence led only to a minor increase of total volume activity of CYP3A4 but lead to an accumulation of inactive CYP. Translocation efficiency is therefore probably not the limiting factor for more efficient cell-free CYP production. Future studies may identify the remaining restrictions thereby increasing the amount of holo CYPs.

One of the main goals of cell-free CYP synthesis is the development of a screening system^41^, allowing the parallel analysis of different CYPs. As a proof of principle the human CYP1A2, and CYP2B6 were synthesized showing the straightforward expandability of the cell-free system to CYPs from other gene families. With 10% (CYP1A2), 5% (CYP2B6) and 20% (CYP3A4) participation in CYP metabolism, these CYPs are among the most important representatives of their respective gene families in research and industry^5^. Transcriptome data suggests, that no homologs of these three human CYPs are expressed in CHO cells^57^. Accordingly, as for CYP3A4, no significant activity of the other CYPs was measured in lysates of parental CHO or CHO-CPR before the CYP synthesis. The absence of a background CYP activity also demonstrates for CYP1A2 and CYP2B6 how well the CHO cell-free system is suited for specific CYP synthesis and thereby for the generation of mono-CYP microsomes.

The turnover of pharmaceutically relevant CYP substrates by cell-free produced CYPs could be detected indirectly, by analyzing the competitive turnover in the luciferase substrate-based CYP assays. Interactions of a tested CYP with defined substances results in a change in the luciferase assay activity, which was observed here for all three CYPs for several known substrates/substances.

The sterol hormone testosterone is probably the best studied substrate especially concerning CYP3A4^58,59^. Interactions of CYP3A4 with midazolam and efavirenz^60,61^ were also confirmed during the assay. Similar to CYP3A4, several substrates influenced the activity of CYP1A2. This is also in accordance with previous studies^62^ and confirms the successful cell-free synthesis of this CYP isoform. CYP2B6 activity was influenced by efavirenz, a well-known CYP2B6 substrate^63^. In contrast to other substrates, the CYP substrate testosterone had an activity increasing effect on CYP2B6. This atypical kinetic characteristic of substrate activation by testosterone has already been observed earlier for CYP2B6, due to autoactivation of the enzyme^64,65^. Upon initial inspection, an anomaly in the assay appears to be present. However, as the values exhibited reproducibility, it was inferred that this discrepancy is attributed to an autoactivation of the enzyme upon substrate binding, resulting in increased Luc substrate turnover.

## Conclusion

The high demand of active CYPs requires a straightforward method for the synthesis of members of this enzyme superfamily. Cell-free protein synthesis enables the synthesis of specific active CYPs using a timesaving procedure. By creating a vesicle containing protein production platform from modified CPR overexpressing CHO cells, the generation of mono-CYP microsomes for a huge variety of future applications becomes feasible. However, this synthesis methodology represents a technological innovation in the field of the production of membrane-attached enzymes. Consequently, there is still a huge potential to be addressed, especially regarding the optimization of the translocation process. So far, it was already possible to use cell-free synthesized CYPs for analytical set-ups. Extensive screening procedures regarding mutations, isoforms and genetic variants, but also detailed substrate and inducer/inhibitor screenings are now facilitated by using CFPS. These promising initial results can be a starting point for various fundamental and applied research projects.

### Supplementary Information

Supplementary Figures.

## Data Availability

All data generated or analyzed during this study are included in this published article (and its Supplementary Information files).
