# Supplementary material for: Synthesis of mono Cytochrome P450 in a modified CHO-CPR cell-free protein production platform
Source: Sci Rep. 2024 Jan 13;14:1271. doi: 10.1038/s41598-024-51781-6 (PMC10787779; doi:10.1038/s41598-024-51781-6)
Supplement: Supplementary file 1 — Supplementary Figures. [file 41598_2024_51781_MOESM1_ESM.pdf]

Synthesis of mono Cytochrome P450 in a modified CHO-CPR cell-free protein production platform

Jan Felix Knauer[1,2,3], Christian Schulz[1,4], Anne Zemella[2], Doreen A. Wüstenhagen[2], Ruben Magnus Walter[2,5], Jan-Heiner Küpper[4], Stefan Kubick[2,3,6]

[1]Fraunhofer Project Group PZ-Syn of the Fraunhofer Institute for Cell Therapy and Immunology, Branch Bioanalytics and Bioprocesses (IZI-BB), Potsdam, Germany, [2]Fraunhofer Institute for Cell Therapy and Immunology (IZI), Branch Bioanalytics and Bioprocesses (IZI-BB), Potsdam, Germany, [3]Freie Universität Berlin, Institute of Chemistry and Biochemistry – Biochemistry, Berlin, Germany, [4]Institute of Biotechnology, Brandenburg University of Technology Cottbus-Senftenberg, Senftenberg, Germany [5]Institute of Biotechnology, Technische Universität Berlin, Gustav-Meyer-Allee 25, 13355, Berlin, Germany [6]Faculty of Health Sciences, joint Faculty of the Brandenburg University of Technology Cottbus – Senftenberg, the Brandenburg Medical School Theodor Fontane and the University of Potsdam, Potsdam, Germany. *Anne.Zemella@izi-bb.fraunhofer.de

Supplementary

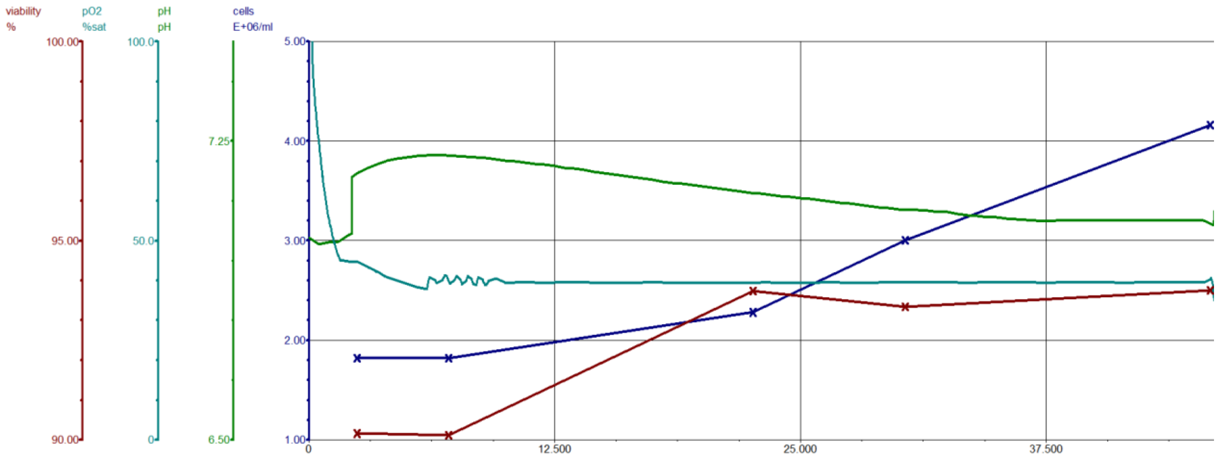

Figure A 1: Growth of CHO-CPR cells in a 5 L fermenter in suspension. The cells were fermented in serum-free ProCHO5 medium (Lonza) at 37°C and 5% CO_2. The cells were cultured while pH and oxygen supply were kept constant until a cell density of. 4 × 10^6 cells/ml was reached. During fermentation the parameters stirrer speed, pH, pO_2 and cultivation temperature were tracked over time and cell counts were measured regularly. The different colored lines show the growth density (blue) the viability (red) the oxygen level (turquoise) and the pH (green) over the fermentation time of 48 h.

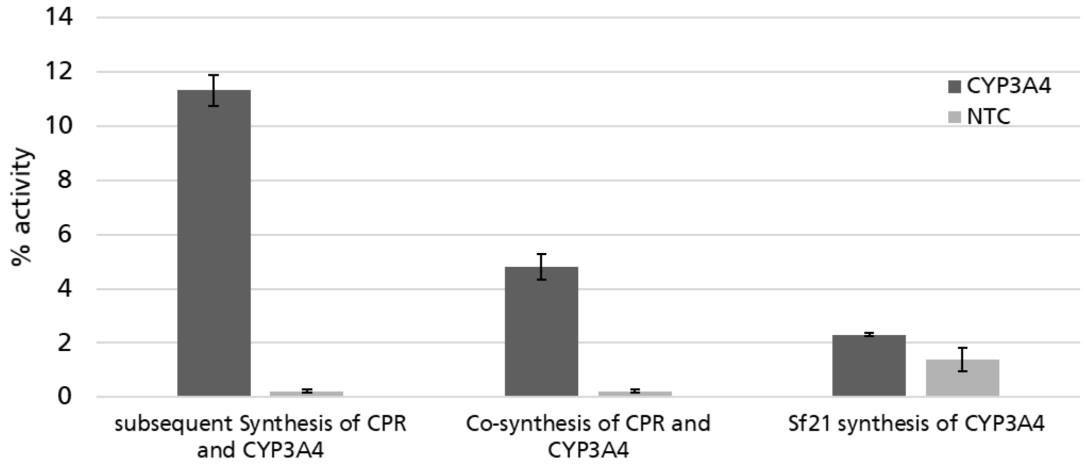

Figure A 2 A) Synthesis of CYP3A4 in three different synthesis formats. Activity of CYP3A4 is given as percentage of the activity of CYP3A4 produced in CHO-CPR lysate. CYP3A4 was produced in a repetitive synthesis using CHO-WT lysate, where in a first synthesis of CPR was produced. Afterwards the microsomes were were separated from the reaction mix and resuspended in second reaction mix for the subsequent synthesis of CYP3A4. Alternatively, both proteins were synthesized at the same time in a co-synthesis in CHO-WT lysate. Thirdly a synthesis in Sf21 lysate was carried out (n = 3).

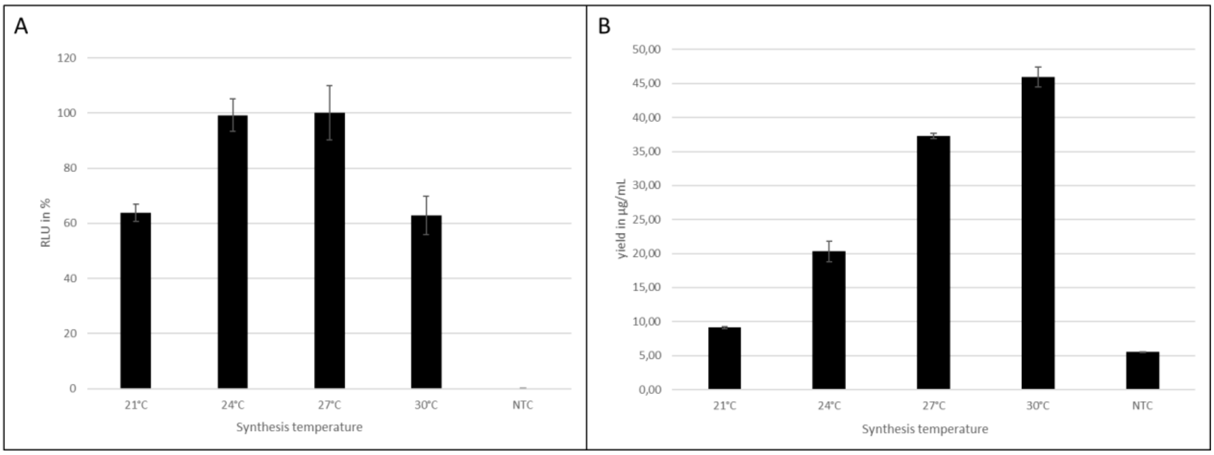

Figure A 3 A) Synthesis temperature screening of CYP3A4. After cell-free synthesis of CYP3A4 in the modified CHO-CPR-lysates for 3 h at different temperatures, the enzyme activity was determined by an IPA-luciferase assay (Promega). B) Additionally, the yield of cell-free produced proteins was determined via radioactive labeling followed by TCA precipitation and scintillation counting. Standard deviations were calculated from triplicate analysis (n = 3).

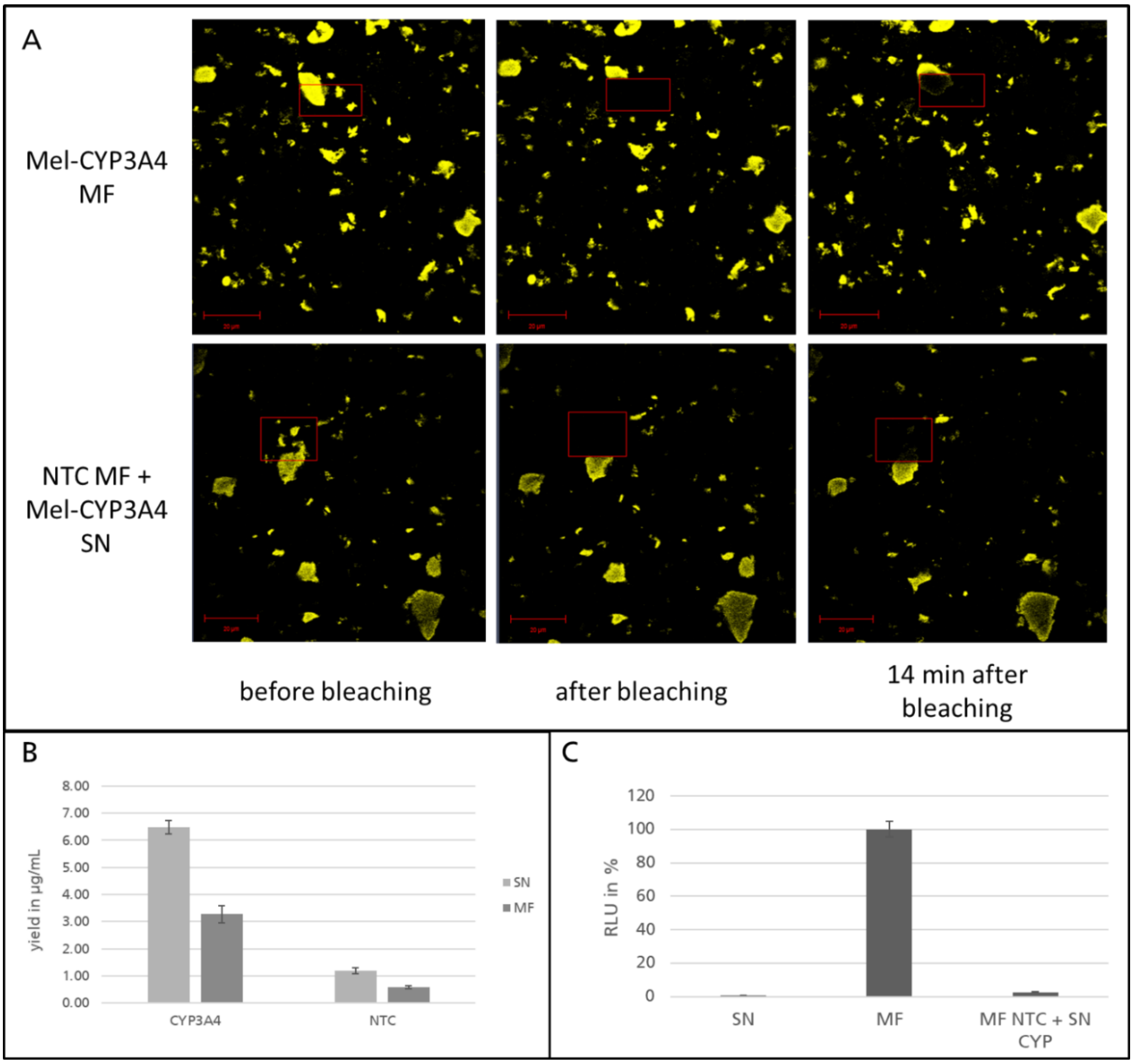

Figure A 4 A) Confocal microscopy images of cell-free synthesized Mel-CYP3A4-eYFP. Proteins were synthesized in the batch mode for 3 h. The microsomal fraction was analyzed and compared to the microsomal fraction of an NTC where the supernatant fraction of the CYP batch was added. Photo bleaching of the region in the red rectangle was photo bleached. The images before after and 14 min after photo bleaching are depicted. B) Yield determination of cell-free produced proteins via radioactive labeling followed by TCA precipitation and scintillation counting. C) Relative activity of CYP3A4 in the supernatant fraction (SN), the microsomal fraction (MF) and in the microsomal fraction of an NTC to which the supernatant fraction of a 3 h CYP batch synthesis was added and incubated for about an hour. 3 µL per well of the samples were applied in the assay Standard deviations for B and C were calculated from triplicate analysis (n = 3).

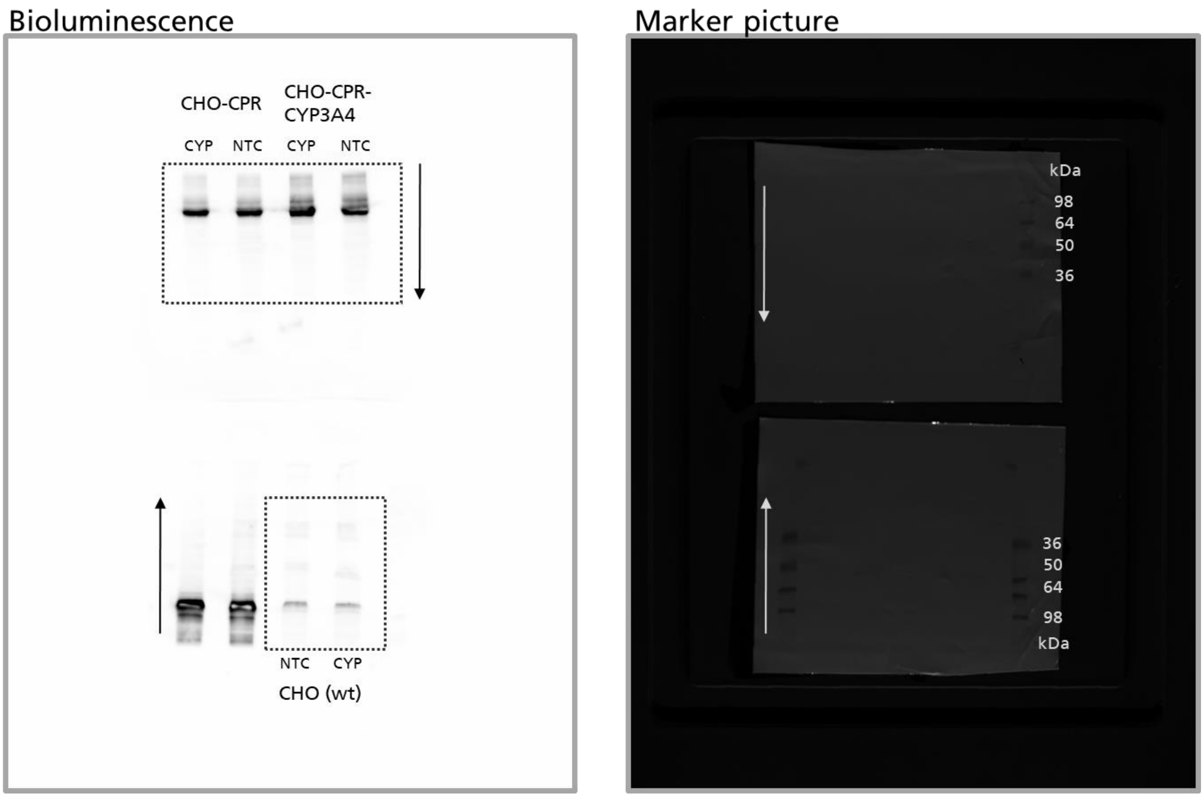

Figure A 5: Full-length blot from figure 2 a. Left panel shows the detected luminescence, the right panel shows a photograph of the blots including the marker. Arrows indicate direction of the gel-run. Dotted boxes indicate lanes that are represented in figure 2 a.

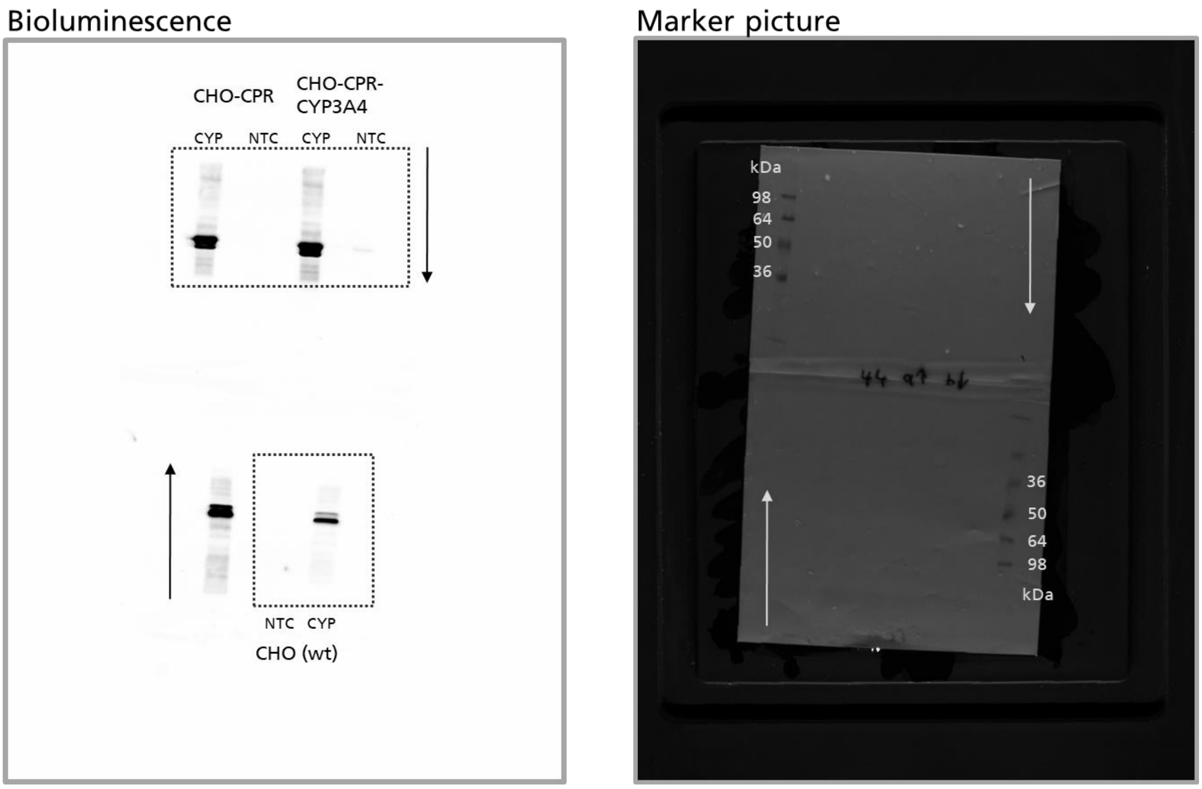

Figure A 6: Full-length blot from figure 2 b. Left panel shows the detected luminescence, the right panel shows a photograph of the blots including the marker. Arrows indicate direction of the gel-run. Dotted boxes indicate lanes that are represented in figure 2 b.

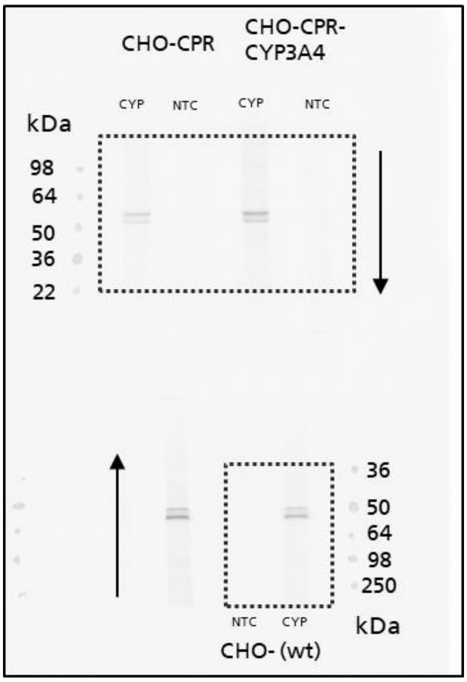

Figure A 7: Full-length autoradiograph from figure 2 c. Arrows indicate direction of the gel-run. Dotted boxes indicate lanes that are represented in figure 2 c.
